# Application and Development of Biocontrol Agents in China

**DOI:** 10.3390/pathogens11101120

**Published:** 2022-09-29

**Authors:** Jiaxuan Meng, Xiuyu Zhang, Xingshan Han, Ben Fan

**Affiliations:** Co-Innovation Center for Sustainable Forestry in Southern China, College of Forestry, Nanjing Forestry University, Nanjing 210037, China

**Keywords:** biocontrol agent, microbial fertilizer, China

## Abstract

While the growing population in the world has a large demand for food, agriculture and forestry are currently facing severe challenges due to phytopathogens and pests along with global warming. For half a century chemical pesticides and fertilizers have made a great contribution to agricultural production. However, the excessive use of chemical agents has caused obvious side effects on the environment and the sustainable development of agriculture in the long term. China has recorded one of the fastest economic growths for more than 20 years but at the cost of a seriously polluted environment. Since a decade ago, China has paid increasing attention to environment protection and taken intensified measures for pollution control and ecological restoration. In this context, the biocontrol agent industry in China has experienced a golden decade of rapid development. In this minireview, we will introduce the application and development of microorganism-based biocontrol agents in China over the past two decades.

## 1. Introduction

China has been a large agricultural country for thousands of years with a long-held notion that sufficient food supply should always be the most important national issue. In the past century, the large-scale application of chemical fertilizers and pesticides has greatly increased global food production, but it has also brought thorny ecological problems and environmental pollution to the world as well as to China. Therefore, finding alternatives to partly replace the traditional chemical fertilizers and pesticides is extremely important for sustainable agricultural development and environmental protection. Due to the prominent advantages, the use of microorganisms as an alternative is one of the most promising solutions. 

Biocontrol agents usually refer to beneficial microorganisms that can control plant diseases through their activities, mainly by killing or reducing the number of plant pathogens. In ecological essence, biocontrol agents use intraspecific or interspecific struggles in the same ecological niche to inhibit the survival and life activities of pathogens. The industrial production of biocontrol agents in China can be mainly grouped into two types: microbial pesticides and microbial fertilizers. Here, we briefly review the application and development of biocontrol agents in China over the past two decades with a focus on microbial fertilizers with biocontrol effects.

## 2. Microbial Pesticides (MPs)

MPs are living bacteria, fungi, viruses, or protozoa that can directly kill agricultural pests to avoid crop damage [1]. As of November 2021, 542 microbial pesticide products have been registered in China [2], including those made from bacteria such as *Bacillus thuringiensis* (Bt), *B. subtilis*, and *Metarhizium anisopliae*, fungi such as *Trichoderma* spp., *Verticillium chlamydosporium*, and *Conidioblous thromboides*, viruses such as cockroach virus, *Pieris rapae* granulovirus (PiraGV), and *Helicoverpa armigera* nucleopolyhedrovirus (HearNPV), and protozoa such as *Paranosema locustae* (*Microsporidia*) [2].

At present, MPs have been applied in China for the biocontrol of various agricultural and forestry diseases or insect pests. For example, it was reported that *Mamestra brassicae* NPV suspension, Bt wettable powder (WP) or suspension concentrate (SC) and *Empedobacter brevis* suspension were used for the control of *Spodoptera frugiperda* larvae in maize, with a biocontrol efficacy of 53.45%, 53.22%, and 52.81%, respectively, after 14 days [3]. The wettable powder of a *Bacillus* endophyte can effectively control root rot of the red kidney bean (*Phaseolus vulgaris* L) with a biocontrol efficacy of 45.47% after 30 days, which is not significantly different from chemical pesticide treatment [4].

The biocontrol efficacy of Bt is quite stable and reliable, and has been widely used in different countries for more than 70 years since World War II. Bt products occupy first place in the Chinese MP market shares, having been applied to 10 million acres of land [5]. China can produce high-quality WP and SC products of Bt, which have a similar virulence efficacy and biocontrol effect to other international products. China has also established its own titer technology system for Bt virulence using the cotton bollworm and diamondback moth as model insects.

## 3. Microbial Fertilizers (MFs)

**Figure 1:**
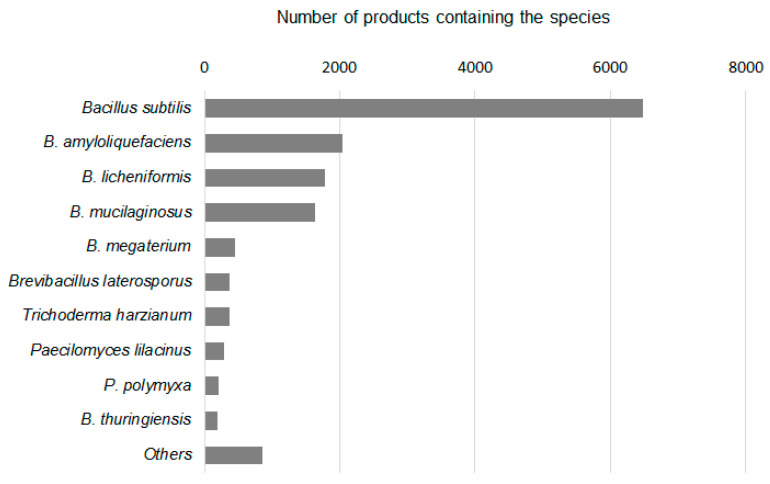
The top 10 microbial species used in microbial fertilizer products in China. Note that many products contain more than one microbial species.

MFs are a kind of fertilizer containing living microorganisms, which can promote plant growth, increase the quality of agricultural products, and/or improve plant stress resistance [6,7,8]. The development and application of MFs in China has a history of nearly 80 years. In the 1960s, rhizobium inoculants to soybean, alfalfa, and vetch were put into practice in China on a large scale [9]. Nowadays, MFs in China have developed into a wide variety of products with diverse functions [9,10,11]. However, the latest statistics show that 93% of MF products in the present Chinese market are made from or at least contain *Bacillus* species, with *B. subtilis* accounting for 67% and *B. amyloliquefaciens* accounting for 21% of the products [12] (Figure 1). It is well known that both species have a strong biocontrol effect against many phytopathogens. Since the registration as an MP is subject to a strict procedure, many manufactures sell their products with biocontrol effect as microbial fertilizers. This is why we consider MFs in China as a type of biocontrol agent and review them here. 

### 3.1. Classification of MFs

Microbial fertilizers in China are officially categorized into three groups: microbial inoculants, bio-organic fertilizers and compound microbial fertilizers [11]. Microbial inoculants refer to liquid living bacteria obtained by fermentation, or solid products made by adsorbing a beneficial microorganism onto a sterile carrier. Microbial inoculants usually impose their beneficial effects on plants via their metabolic activities, but not by directly providing nutrients. At present, there are 10 types of microbial inoculants in China: rhizobium inoculant, nitrogen-fixing bacteria, phosphate-dissolving bacteria, silicate-solubilizing bacteria, photosynthetic bacteria, plant-growth-promoting bacteria, mycorrhizal inoculant, compound microbial inoculant, organic decomposer, and soil-remediation bacteria [13,14].

Bio-organic fertilizers refer to a mixture of animal and plant residues (such as livestock and poultry manure, crop straw, etc.), which are first detoxified and decomposed, and then supplemented with beneficial microorganisms. Bio-organic fertilizer combines the advantages of organic fertilizers and living microbial inoculum, providing plants with both organic matter and beneficial living microorganisms. Bio-organic fertilizer is a kind of long-lasting and environmentally friendly fertilizer, because it recycles agricultural waste and thus reduces environmental pollution.

Compound microbial fertilizers define those fertilizers that are simultaneously composed of organic fertilizers, beneficial microorganisms, and other additions such as trace elements. Compound microbial fertilizers possess the advantages of all the single-variety fertilizers. Microbial inoculants, bio-organic fertilizers, and compound microbial fertilizers account for 52%, 28.4%, 19.5% of the quantity share of China’s microbial fertilizer products, respectively [12].

### 3.2. Application and Development of Microbial Fertilizers

**Figure 2:**
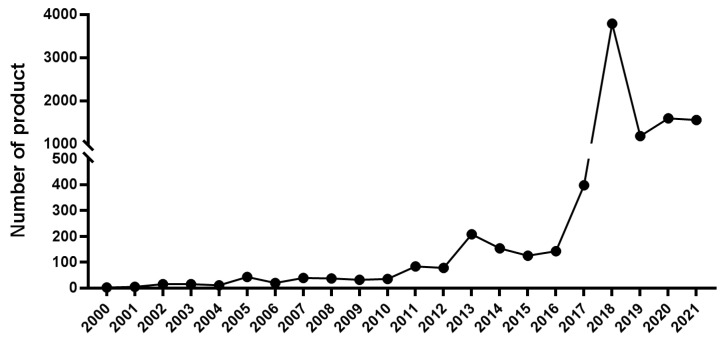
Registered microbial fertilizer product in China from 2001 to 2021.

In the past two decades, China’s microbial fertilizer industry has experienced its golden time of rapid development. During the first ten years of the 21st century, only a limited number of MF products were registered each year (Figure 2). In 2006, there were around 500 MF manufacturers in China with an output of 5 million tons and 498 commercial products [13]. Since 2011, the development of the industry has begun a fast track (Figure 2). Until 2017, there had been as many as 6068 MF enterprises, with nearly 3500 products registered and an annual output of more than 12 million tons [14]. Up to the end of 2021, a total of 9563 commercial products have been registered by more than 2000 manufacturers. The production capacity in 2020 was as high as 24 million tons, being applied to a 2 × 10^8^ h.a. area of land [15]. In an overview, the MF production peaked in 2018, when the number of registered products began to drop but still remained at a high level compared to the previous ten years.

MFs are not only produced and used on a large scale in China; some of them, such as silicate-solubilizing bacteria, compound microbial inoculants and bio-organic fertilizers, have entered the international market, including Australia, India, Thailand and other countries [16].

The fast development of the MF industry in China is connected to the Chinese governmental support of related policies. China has intensified its support for the basic research and application promotion of bio-fertilizers over the last 20 years. The project “Key Technology Research and Industrialization Promoting Program on Developing New Slow- and Controlled-Releasing Fertilizers and Organic Fertilizers” was established in the 12th Five-Year Plan of China. The project “Creation and Production of Microbial Agents and Enzyme Products for Agricultural Purpose” was implemented in the National Science and Technology (863) Program of China [17]. In addition, China also launched a series of complementary programs such as the Soil-Characterization-Based Fertilizer Application Program, the Arable Land Protection and Quality Improvement Program, and the Program for Remediation of Heavy-Metal-Polluted Soil in Hunan [11]. 

Meanwhile, a series of promotion measures were put into practice in order to directly impel the application of organic/microbial fertilizers. For instance, in 2001, the General Administration of Quality Supervision, Inspection and Quarantine of China issued safety standards for vegetables, fruits, livestock and poultry meat, aquatic products, and corresponding environmental standards for their production areas, which legally allow organic fertilizers and MFs to be used as the main fertilizer variety for their production. In 2015, the former Ministry of Agriculture of China released two policies: “the Action Program for Zero Growth in Chemical Fertilizer Use till 2020” and “the Action Program for Zero Growth of Chemical Pesticide Use till 2020” as an instruction for pushing the replacement of chemical fertilizers/pesticides with organic and biological fertilizers [11,18]. Moreover, “the Action Program for the Replacement of Chemical Fertilizers with Organic Fertilizers on Plantation of Fruit Trees, Vegetable, and Tea” was released in 2017 [19] and “the Technical Guidance on the Replacement of Chemical Fertilizers with Organic Fertilizers on Plantation of Fruit Trees, Vegetable, and Tea” was released in 2020 [20]. Altogether, these programs or measures significantly facilitated the progress of the MF industry in China.

### 3.3. Effects of Application of MFs

Many scientific reports, some in Chinese, have recorded the effects of the application of MFs in China. First of all, MFs have been widely applied to a variety of staples such as maize, rice, and wheat, as well as main vegetables consumed in China such as tomato, lettuce, and cucumber. They can directly stimulate the growth of these plants. An application of microbial inoculants producing polyglutamic acid recorded a significant improvement in maize yield by 21.17% compared with the chemical fertilizer control [21]. Another application of an MF compound enabled a cucumber yield of 3865.5 kg per 667 m^2^, which was 252.5 kg more than the conventional fertilization [22]. 

MFs have also revealed their good biocontrol effects on many plants. In contrast to numerous reports on the biocontrol effect of a single bacterial inoculant, the biocontrol effects of bio-organic fertilizers have also been intensively investigated in China. Rhizosphere bacteria and derived bio-organic fertilizers have been evaluated as potential biocontrol agents against pathogens that cause bacterial wilt (*Ralstonia solanacearum*) in potato, showing their remarkable biocontrol efficacy in addition to the promoting effect on the potato yield [23]. The potent efficacy of bio-organic fertilizers against tobacco bacterial wilt, cucumber Fusarium wilt, ginger rhizome rot, tomato bacterial wilt, cotton verticillium wilt, etc. have been reported [24,25,26,27,28]. Plant disease suppression by a bio-organic fertilizer not only relies on its own beneficial microorganism but also involves mobilizing beneficial indigenous soil bacteria [29,30]. In an elegant study, Deng et al. reported that bio-organic fertilizer could promote the suppression of *R. solanacearum* by inducing changes in the functionality and composition of rhizosphere bacterial communities [31]. 

In general, MFs have positive effects on plant yield and quality through a direct stimulation mechanism or an indirect biocontrol effect, which has been widely recognized by the Chinese population. This is an important factor for the commercial success of MFs in China and lays a substantial foundation for their high-quality development in the future.

## 4. Outlook

Biocontrol agents are of great value in reducing plant pests and diseases, and thus promote agricultural production. Meanwhile, biocontrol agents reduce the use of chemical pesticides and thus protect the environment. With the advancement of microbial technology, the biocontrol industry in China is making a continuous progress; the MF industry has especially experienced a bloom in the past two decades, leading the world at least in terms of production and application scale. However, there are still many problems restricting the further development of the industry in China. For example, basic research on the mechanisms of microbial action is still insufficient, the stability of many agents in the field have not been well resolved, and new forms of agents for easy application should be created. In the field of industrial production, there are also many problems such as immature business management, varied product quality and backward equipment in some manufacturers. Therefore, China still requires a lot of improvement in this industry. In the follow-up development stage, China should pay more attention to product quality than to product number and application scale, which can then provide greater help for the sustainable development of Chinese agriculture.
